# 산후출혈 산모 간호 시뮬레이션 교육 프로그램의 효과

**DOI:** 10.4069/kjwhn.2020.03.04

**Published:** 2020-03-17

**Authors:** Miok Kim, Juyoung Ha

**Affiliations:** 1 Department of Nursing, Tongmyong University, Busan, Korea; 1 동명대학교 간호학과; 2 College of Nursing, Pusan National University, Busan, Korea; 2 부산대학교 간호대학

**Keywords:** Education, Postnatal care, Postpartum hemorrhage, Simulation training, 교육, 산후간호, 산후출혈, 시뮬레이션 교육

## Abstract

**Purpose:**

This study was conducted to develop a simulation-based postpartum care education program for women with postpartum hemorrhage and to verify the effects of the program on postpartum care.

**Methods:**

This program was developed according to the ADDIE model of instructional system design, which consists of analysis, design, development, implementation, and evaluation phases. This quasi-experimental study used a non-equivalent control group pre- and post-test design, and data were collected from April 23 to May 4, 2015. To verify the effects of the program, 33 nursing students in the experimental group participated in a simulation program, whereas 31 students in the control group were given a case study.

**Results:**

The experimental group had statistically significantly higher scores for clinical performance (t=–4.80, *p*<.001), clinical judgment (t=–4.14, *p*<.001), and learning satisfaction (t=–10.45, *p*<.001) than the control group.

**Conclusion:**

The results of this study indicate that the simulation-based postpartum care education program for women with postpartum hemorrhage was effective for developing students’ competency, implying that a similar program should be integrated into the clinical training component of the maternal nursing curriculum.

## Introduction

### 연구 필요성

간호교육에서 임상실습교육은 총체적인 간호지식과 실무를 통합할 수 있는 기회를 제공하며 의사소통과 간호술의 적용, 문제 해결, 의사결정 등 전문직 간호사로서의 기본능력을 갖도록 한다[1]. 그러나 급박하고 예측할 수 없는 임상환경과 대상자의 의식 증가 및 현장 교육인력의 부족으로 인해 실제 실습교육은 주로 관찰 위주로 진행되고, 간호교육기관의 양적 증가에 따른 실습기관의 부족으로 효율적인 실습교육이 이루어지기 어려운 실정이다. 특히 여성건강간호학 임상실습의 경우 한 병원당 1–2개의 병동 단위 분만실과 산부인과에서 이루어지기 때문에 실습지 확보에 어려움이 따르고 낮은 출산율과 맞물려 실습 대상 사례가 부족하다[2]. 또한 분만실은 병원 이용자의 인권 존중과 사생활 보호에 특히 민감한 특수 환경으로, 대상자 접근과 간호중재 제공이 어렵고 술기 수행의 기회까지 감소하게 된다[3]. 그럼에도 불구하고 실제 병원 환경에서는 간호 대상자의 중증도와 응급수준이 다양화되고 있는 상황이므로 일정 수준의 임상수행능력을 갖추고 복잡한 역할을 수행할 수 있는 비판적 사고와 임상판단력을 갖춘 졸업 간호사를 필요로 하고 있다[4].

임상현장의 요구와 임상실습교육의 제한점을 보완하여 학생들의 간호 역량을 향상시키는 교수 학습 전략으로 반복학습, 피드백, 평가와 성찰의 기회를 제공하며, 실제의 재현, 적극적인 학습자 참여 촉진, 실무와 이론학습 간의 통합을 포함하는 시뮬레이션을 활용한다[5]. 시뮬레이션 학습은 또한 학생들로 하여금 지식과 기술을 연계하면서 통합적이고 비판적인 임상적 판단 능력과 문제 해결 능력을 갖게 한다[6]. 최근 고연령, 고위험 산모의 증가에 따라 분만실과 산과병동에서 산후출혈 산모 간호의 중요성이 높아지고 있는데, 이는 간호사의 즉각적인 처치가 필요한 임상적 응급상황이다. 그러므로 간호사는 산후출혈에 대한 사정 기술과 상황 판단력 및 적절한 간호중재를 위한 비판적 사고능력과 임상수행능력을 갖추어야 한다.

간호학생을 대상으로 시뮬레이션 교육 효과를 검증한 선행연구에서 실제 실습에서 경험하기 어려운 응급상황 관리에 있어 시뮬레이션 교육이 효과적이었으며[7,8] 전통적 교육방법에 비해 학업성취도가 유의하게 향상됨을 확인하였다[9]. 따라서 산욕기 간호상황 중 응급상황인 산후출혈에 관한 시뮬레이션 기반 교육 프로그램을 개발하고 간호학생에게 적용하여 그 효과를 검증하는 것이 필요할 것이다. 더불어 시뮬레이션 교육 효과에 관한 국내 선행연구 대부분이 지식, 수행능력, 수행 자신감, 자기 효능감에 대한 자가보고 자료를 분석하였으므로, 적절한 문제 해결에 필요한 임상간호 수행능력과 더불어 비판적 사고능력 및 임상판단력에 대해 객관적인 평가를 실시해 볼 필요가 있을 것이다. 이에 본 연구에서는 학습자의 활동을 촉진시켜 학습효과를 극대화하는 조직적인 절차와 과정인 교수체제설계[10]에 따라 산후출혈 산모 간호 시뮬레이션 교육 프로그램을 개발하고 그 효과를 검증하고자 한다.

### 연구 목적

본 연구의 목적은 산후출혈 산모 간호 시뮬레이션 교육 프로그램을 개발하고 이것이 간호대학생의 임상수행능력, 비판적 사고성향, 임상판단력에 미치는 효과를 파악하는 것이다.

## Methods

Ethics statement: This study was approved by the Institutional Review Board (IRB) of Pusan National University (IRB/2014-73-HR). Informed consent was obtained from the subjects.

본 연구는 교수체제설계모형[10]에 따라 분석, 설계, 개발 단계를 통해 산후출혈 산모 간호 시뮬레이션 교육 프로그램을 개발하고, 실행, 평가 단계에서 비동등성 대조군 전후 실험설계를 실시하여 개발된 프로그램의 효과를 검증하였다.

### 산후출혈 산모 간호 시뮬레이션 교육 프로그램 개발 절차

#### 분석단계

분석단계에서는 교육 요구, 학습과제, 학습자, 학습환경을 분석하였다.

학습자의 교육 요구는 김해대학 간호학과 3학년에 재학 중인 남녀 학생 69명을 대상으로 산후출혈 산모 간호 시뮬레이션 교육의 필요성, 희망 실습항목, 희망 교육내용과 교육 후 기대하는 결과에 대한 설문조사로 확인하였다. 응답자의 94.2%가 산후출혈 산모 간호 시뮬레이션 교육이 필요하다고 하였고, 희망 실습항목은 출혈 원인 사정(94.2%), 양상 사정(88.4%), 출혈 중재(87.0%) 순이었으며, 희망 교육내용은 분만 후 출혈 사정(82.6%)와 분만 후 자궁 사정(75.4%) 순이었으며, 교육 후 실제 간호상황에의 적용 기대가 48.4%로 가장 높게 나타났다.

학습과제의 타당성은 시뮬레이션 관련 문헌[9,11]을 검토한 결과, 반복훈련과 긍정적 학업 성취를 위해 ‘산후출혈’에 관한 시뮬레이션 교육이 필요함을 확인하였다. 학습자 특성은 간호학 전공의 3학년 학생으로 30병상 이상의 3개 여성전문병원 중 한 곳에서 한 학기 동안 여성건강간호학 임상실습을 2학점 이수한 경험이 있으며, 시뮬레이션 실습교육 경험은 없음을 확인하였다.

학습환경은 김해대학 간호학과 시뮬레이션 실습실의 현장조사를 통해, 학생들의 실습공간과 통제실이 분리되어 있어 효과적인 시뮬레이션 구현과 학생들의 실습활동 모니터링이 가능하고, 빔 프로젝터, 컴퓨터, 스크린이 설치되어 있어 실습 후 디브리핑(debriefing) 시 동영상 시청이 가능하며, 조별학습을 위한 책상과 의자가 비치되어 있음을 확인하였다. 실습공간은 산부인과 병동의 병실 구조로, 시뮬레이션 수업 운영에 필요한 SimMom 모형(Laerdal Medical Corp., Gateville, TX, USA)을 비롯한 시청각 장비, 시나리오 운영을 위한 소프트웨어 및 하드웨어를 갖추었으며, 비기술적 장비로 혈압계, 체온계, 청진기, 윤활제, 산모패드, 수액, 약물, 주사기, 주사바늘, 정맥수액세트, 앰플 등의 소모품이 준비되어 있었다.

#### 설계단계

설계단계에서는 교육목표와 교육내용을 설정하고, 교육운영방법 설계, 교육매체 선정, 평가도구 선정을 진행하였다. 먼저, 한국간호과학회의 모성간호학 학습목표[12]와 한국간호교육평가원[13]에서 제시한 12개의 학습성과를 바탕으로 ‘산후출혈을 이해하고 대상자에게 간호과정을 적용하여 산모 간호를 수행할 수 있다’로 교육목표를 설정하고 학습단계별로 구체적인 학습목표를 설정하였다. 교육내용은 전국 간호대학에서 사용빈도가 높은 여성건강간호학 교재 3종[14-16]과 관련 문헌[17-20]을 고찰하여 산후출혈의 정의, 산후출혈의 원인, 간호사정, 치료와 간호중재의 순서로 제목을 분류하였고, 산후출혈의 원인 중 발생빈도가 높은 자궁이완, 산도열상, 잔류태반에 중점을 두어 원인에 따른 간호사정, 간호중재를 소제목으로 분류하여 내용을 구성하였다.

교육운영은 이론수업을 통한 사전학습과 시뮬레이션 실습으로 설계하였다. 이론수업은 강의실에서 산후출혈 산모 간호에 관한 이론수업용 학습자료를 이용하여 2시간 동안 진행하고, 시뮬레이션 실습교육은 초기 15분 동안 시뮬레이터에 대한 오리엔테이션과 시나리오 제공, 조별로 15–20분 동안 시뮬레이션 실습, 실습 후 전체 학생을 대상으로 40분간 디브리핑의 순서로 총 2시간 진행하는 것으로 하였다.

교육매체는 이론수업용 학습자료, 학생들이 기록하는 시뮬레이션 실습활동 기록지, 시뮬레이터, 오디오/비디오 시스템, 실습 기자재를 선정하였다. 교육 효과에 대한 평가도구는 임상수행능력, 비판적 사고성향, 임상판단력 도구를 선정하였다. 이는 Jeffries의 simulation model [21]에서 제시하는 지식과 간호수행을 지식과 기술을 통합적으로 평가하는 임상수행능력으로, 비판적 사고는 비판적 사고성향과 임상판단력으로 평가하고자 하였으며, 본 연구에서는 교육만족도 평가를 추가하였다.

#### 개발단계

개발단계에서는 교수자료를 개발하고 내용타당도 검정을 실시하였다. 교수자료로 산후출혈 이론수업용 학습자료, 시나리오, 시뮬레이션 실습활동 기록지를 개발하였다. 이론수업용 학습자료는 산후출혈 산모 간호에 대한 유인물과 프레젠테이션 자료를 제작하였다. 시나리오는 자궁이완, 산도열상, 잔류태반 상황으로 구분하여 3개를 개발하였고, Jeffries의 simulation model [21]에서 제시된 교육적 실무요소와 시뮬레이션 설계요소에 근거하여 산후출혈의 공통 사정 및 중재내용과 각 원인별 사정 및 중재내용으로 나누어 구성하였다. 시나리오 구성 절차는 미국간호연맹에서 제안한 형식을 토대로 개요, 알고리즘, 교수용 평가 체크리스트, 디브리핑 계획으로 구성하였다. 실습활동 기록지는 산후출혈 환자의 상태 확인을 위한 체크리스트 형태의 간호사정 기록지와 간호 중재내용을 기록하는 간호 기록지, 투약 기록지, 그리고 디브리핑 가이드라인에 근거한 성찰일지로 구성하였다.

개발된 교육 프로그램에 대하여 여성건강간호학 교수 2인, 대학병원 분만실 수간호사 1인, 대학병원 분만실 10년 이상 경력의 간호사 1인, 조산사이며 시뮬레이션 수업을 진행하고 있는 시간강사 1인을 대상으로 내용타당도 조사를 실시하였다. 학습목표, 사전학습 내용, 3개의 시나리오, 디브리핑 내용의 6개 항목에 대해 ‘매우 적절하지 않다’ 1점, ‘대체로 적절하지 않다’ 2점, ‘보통이다’ 3점, ‘대체로 적절하다’ 4점, ‘매우 적절하다’ 5점에 응답하도록 하였고, 각 문항의 내용타당도 지수가 0.8 이상임을 확인하였다. 개발된 교육 프로그램의 적용 가능성 점검 및 기술적 보완을 위해 김해대학 시뮬레이션 실습실에서 간호학과 4학년 학생 4명을 대상으로 시범 운영을 실시하였다. 10분 동안 시나리오를 읽고 역할 분담에 대해 논의하였고, 60분 동안 3개의 시나리오에 대한 시뮬레이션 실습을 진행하였다. 시범 운영 후 실습실과 조작실 사이의 오디오 볼륨을 적절한 수준으로 조정하고, 학생들의 실습동선을 고려하여 물품을 배치하였다.

### 산후출혈 산모 간호 시뮬레이션 교육 프로그램의 효과 검증

#### 실행단계

##### 연구 설계

본 연구는 산후출혈 산모 간호 시뮬레이션 교육이 간호대학생의 임상수행능력, 비판적 사고성향, 임상판단력에 미치는 효과를 분석하기 위한 비동등성 대조군 전후 실험설계이다.

##### 연구 대상

본 연구의 대상은 간호학과 3학년에 재학 중인 학생으로, 산후출혈 산모간 호에 대한 시뮬레이션 학습경험이 없고, 3년제 간호학과 과정 중 2학년 교과과정을 모두 이수한 학생 중 본 연구의 목적과 절차를 이해하고 연구에 참여하기를 동의한 학생 64명이다. 학생 게시판의 공고를 통해 모집된 실험군과 대조군은 2개 실습분반을 이용하여 임의로 배정하였다. 대상자 수는 G-Power 3.0.10 프리웨어[22]를 사용하여 유의수준 0.05, 검정력 0.80, 효과 크기 0.66일 때[23], 두 집단 평균 비교에 필요한 표본수가 실험군과 대조군 각 30명임을 산출하고, 탈락률을 고려하여 실험군과 대조군 각 33명을 계획하였다. 연구 진행 중 대조군에서 불참 1명, 철회 1명으로 총 2명이 탈락되어 최종 대상자는 실험군 33명, 대조군 31명이었다.

##### 연구 도구

임상수행능력: 임상수행능력 측정도구는 본 연구자가 문헌고찰을 토대로 산후출혈 산모 간호에 필요한 지식/기술영역 11문항(전공지식에 근거한 간호술 적용 5문항, 핵심 기본간호술 적용 2문항, 치료적 의사소통술 활용 1문항, 비판적 사고기반의 임상판단력 적용 3문항)과 태도 영역 4문항으로 총 15문항을 작성한 후 여성건강간호학 전공 교수 4인, 석사과정 중이며 대학병원 분만실 임상경력 10년 이상인 간호사 2인, 조산사이며 대학에서 시뮬레이션 수업을 담당하고 있는 시간강사 1인, 석사과정 중이며 대학병원 임상경력 10년 이상인 임상실습지도자로 구성된 전문가 집단을 통해 내용타당도를 검증 받았다. 각 문항은 ‘매우 적절하지 않다’ 1점에서 ‘매우 적절하다’ 5점으로 응답하도록 하였고 모든 문항의 내용타당도 지수는 0.8 이상이었다. 개발된 15문항 외에 전문가 집단의 의견을 수렴하여 지식/기술 영역의 간호사정 항목 1문항을 추가하여 최종 16문항의 임상수행능력 평가도구를 개발하였다. 각 항목은 완전 수행을 ‘우수’ 2점으로, 불완전 수행을 ‘미흡’ 1점으로, 미수행을 0점으로 평가하며, 점수범위는 최저 0점–최고 32점까지로 총점이 높을수록 임상수행능력이 높음을 의미한다. 본 연구에서 도구의 신뢰도 Cronbach’s α값은 .71이었고 평가자 간 신뢰도 상관계수는 .70이었다.

비판적 사고성향: 비판적 사고성향은 Yoon [24]이 간호대학생을 대상으로 개발한 도구를 저자의 허락 하에 사용하였다. 이 도구는 지적열정 호기심 5문항, 신중성 4문항, 자신감 4문항, 체계성 3문항, 지적공정성 4문항, 건전한 회의성 4문항, 객관성 3문항의 7개 요인 27문항으로 구성된 5점 Likert 척도로, 점수가 높을수록 비판적 사고성향이 높음을 의미한다. 개발 당시 도구의 신뢰도 Cronbach’s α값은 .84이었고 본 연구에서는 .90이었다.

임상판단력: Lasater가 개발한 Lasater Clinical Judgment Rubric [25]을 Lim [23]이 우리말로 번역한 도구를 저자의 허락 하에 사용하였다. 이 도구는 인지, 해석, 반응과 성찰의 4가지 영역 11개 항목으로 구성되어 있으며, 각 항목당 전문가 수준은 4점, 초보자 수준은 1점으로 총점은 최하 11점에서 최고 44점이다. Lim [23]의 연구에서 도구의 신뢰도 Cronbach’s α값은 .96이고 평가자 간 신뢰도는 .80이었다. 본 연구에서 Cronbach’s α값은 .91, 평가자 간 신뢰도 상관계수는 .90이었다.

교육만족도: 교육만족도는 본 연구자가 개발한 학습경험의 유용성 1문항, 교육 프로그램 내용 만족 5문항, 교육 프로그램 효과 4문항의 총 10문항으로 측정하였다. 각 항목은 ‘매우 그렇다’ 4점에서 ‘매우 그렇지 않다’ 1점으로 최소 10점에서 최대 40점까지이며 점수가 높을수록 교육에 대한 만족도가 높음을 의미한다. 본 연구에서 도구의 신뢰도 Cronbach’s α값은 .93이었다.

##### 자료 수집 방법 및 절차

###### 사전·사후조사

**Figure. 1.**
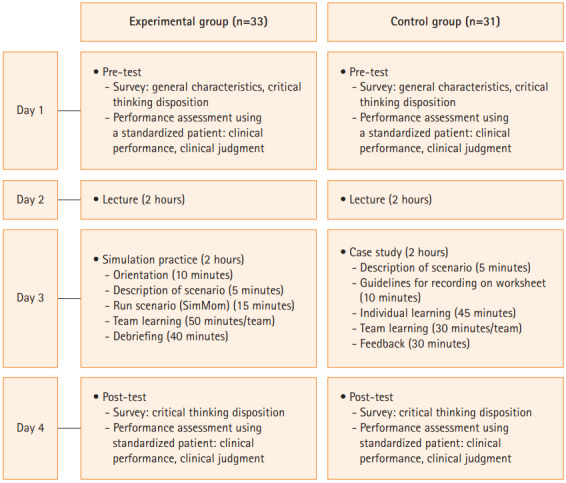
Research process.

실험군과 대조군에게 교육 프로그램 제1일에 사전조사를, 제4일에 사후조사를 실시하였다. 사전, 사후조사 시 설문지를 이용해 비판적 사고성향을 조사하고 표준화 환자를 이용해 임상수행능력과 임상판단력 수행평가를 실시하였다. 사전조사 시 일반적 특성을 확인하였고, 사후조사 시 교육만족도를 확인하였다. 수행평가 시간은 1인당 3분에서 7분 정도 소요되었고 전체 소요시간은 실험군, 대조군 각각 4시간이었다. 연구대상자의 동의 하에 수행평가 과정을 동영상으로 촬영하였고, 촬영된 동영상은 평가자인 연구보조원 2인에게 보내어 평가하도록 하였다(Figure 1).

###### 교육 프로그램 중재

•실험군 중재: 교육 프로그램 제2일에 산후출혈 산모 간호에 대한 이론수업을 2시간 실시하여 자궁이완, 산도열상, 잔류태반에 따른 산후출혈 관련 내용을 모두 학습하고 3가지 원인별 시뮬레이션 실습을 준비할 수 있게 하였다. 제3일에 4–5명으로 구성된 8개의 조별 시뮬레이션 실습을 진행하였는데, 실험군의 모든 학생은 SimMom을 이용하여 실습하였다. 연구자가 시뮬레이션 실습실의 구조와 SimMom의 기능, 물품 사용 등 수업진행 절차에 대해 설명한 후 자궁이완, 산도열상, 잔류태반 중 한 개의 시나리오를 제공하였다. 산후출혈 산모 간호에 있어 자궁이완, 산도열상, 잔류태반 3가지 원인을 하나의 시나리오로 진행할 때는 실습시간이 많이 소요되고 효율성이 떨어질 것을 우려하여 팀별로 한 개의 시나리오를 무작위로 배정하였다. 시나리오 개발 시 공통된 간호사정과 중재 외의 원인에 따른 간호사정과 중재 구성 시에 난이도를 조정하여 시나리오 배정에 따른 차이를 최소화하였다. 각 조의 시뮬레이션은 15분간 구동하였고 교수-학생 상호작용 과정에서 환자에 대한 추가정보를 제공하였다. 실습 후 40분간 3개의 시나리오 시뮬레이션 상황에 대해 디브리핑을 시행하고 실습이 우수한 1개 조의 촬영 비디오를 통해 느낀 점과 보완 사항을 점검하였다.

•대조군 중재: 교육 프로그램 제2일에 실험군과 동일하게 산후출혈 산모 간호에 대한 이론수업을 2시간 실시한 후, 제3일에 3–4명으로 구성된 8개의 조별 사례연구를 진행하였다. 연구자가 수업진행 절차를 설명하고 실험군과 동일한 자궁이완, 산도열상, 잔류태반 시나리오 중 한 개의 시나리오와 환자의 추가정보를 조별로 제공하였다. 개별 학생은 45분간 환자 상황에 따른 간호문제를 도출하고 간호진단을 내린 후, 적절한 간호계획을 세우고 기대하는 평가결과를 사례연구 워크시트에 기록하였다. 이후 30분간 조별 토의를 실시하여 우선 순위에 따른 2개의 간호진단에 대한 간호과정을 적용한 후 최종적으로 조별 사례연구 워크시트에 기록하였다. 연구자는 조별 사례연구 워크시트를 확인하여 수정보완 사항을 점검하였다(Figure 1).

###### 연구보조원 준비와 표준화 환자 훈련

2명의 연구보조원이 수행평가를 실시하였다. 연구보조원 1은 임상경력 20년 이상인 분만실 수간호사로 간호학 석사과정 중이며 여성건강간호학 임상실습 지도에 참여한 경험이 있고, 연구보조원 2는 임상경력 10년 이상인 간호사로 간호학 석사과정 중이며 기본간호학 실습조교로 근무 중이다. 연구보조원 훈련을 위해 연구의 목적과 절차 및 3개의 시나리오에 대한 내용을 설명하고 연구자와 평가자 간 논의를 통해 시나리오에 맞는 구체적 간호내용을 평가할 수 있도록 항목별로 확인하면서 임상수행능력 체크리스트와 임상판단력 평가를 위한 루브릭 평가방법을 훈련하였다.

실험군과 대조군의 임상판단력과 임상수행능력 측정을 위해 4학년 간호학생 2인에게 표준화 환자 훈련을 실시하였다. 현실감을 높이기 위해 적절한 복장과 분장 및 시나리오에 따른 상황을 구현하도록 훈련하였고 실습 학생과 적절히 의사소통할 수 있도록 훈련 대본을 이용하여 3시간 동안 훈련하였다.

##### 자료 분석 방법

수집된 자료는 IBM SPSS Statistics for Windows, ver 20.0 (IBM Corp., Armonk, NY, USA) 프로그램을 이용하여 대상자의 일반적 특성과 각 변수는 빈도와 백분율, 평균과 표준편차를 산출하였고, 대상자의 동질성 검증은 t-test, chi-square test, 또는 Fisher’s exact probability test로, 프로그램 중재에 따른 효과는 t-test로 분석하였다. 측정도구의 신뢰도는 Cronbach’ α coefficient로, 평가자 간 신뢰도는 intraclass correlation coefficient로 검정하였다.

## Results

프로그램 효과의 평가단계에서는 개발된 산후출혈 산모 간호 시뮬레이션 교육 프로그램의 효과에 대해 임상수행능력, 비판적 사고성향, 임상판단력, 교육만족도를 평가하였다.

실험군과 대조군 간 일반적 특성과 사전 임상수행능력, 비판적 사고성향, 임상판단력에 대한 종속변수의 동질성 검증 결과 통계적으로 유의한 차이가 없이 모두 동질한 것으로 나타났다(Table 1). 임상수행능력은 실험군이 사전 5.45±3.86점, 사후 21.38±2.87점으로 15.92점 증가하였고, 대조군은 사전 6.52±4.76점, 사후 16.05±3.49점으로 9.53점 증가하여 두 집단 간에 통계적으로 유의한 차이가 있는 것으로 나타났다(t=–4.80, *p*<.001). 비판적 사고성향은 실험군은 사전 97.18±9.09점, 사후 98.42±11.06점으로 1.24점 증가하였고, 대조군은 사전 98.35±10.35점, 사후 96.77±11.67점으로 1.58점 감소하였으나, 두 집단 간에 통계적으로 유의한 차이가 없었다(t=–1.68, *p*=.097). 임상판단력은 실험군은 사전 18.97±5.67점, 사후 36.38±2.55점으로 17.41점이 증가하였고, 대조군은 사전 19.32±6.97점, 사후 30.39±4.39점으로 11.07점 증가하여 두 집단 간에 통계적으로 유의한 차이가 있는 것으로 나타났다(t=–4.14, *p*<.001). 교육만족도는 실험군은 평균 23.97±2.70점, 대조군은 평균 16.45±3.05점으로 두 집단 간에 통계적으로 유의한 차이가 있는 것으로 나타났다(t=–10.45, *p*<.001; Table 2).

## Discussion

본 연구에서는 시뮬레이션 기반 산후출혈 산모 간호 교육 프로그램을 개발하여 간호학생의 임상수행능력, 비판적 사고성향, 임상판단력에 미치는 효과를 파악하고 교육만족도를 확인하였다. 본 연구에서 산후출혈 산모 간호 시뮬레이션 교육을 받은 실험군은 사례연구를 학습한 대조군보다 임상수행능력이 향상되었다. 이러한 결과는 간호대학생을 위한 시뮬레이션 교육 프로그램의 효과를 검증한 선행연구[23]의 결과와 유사하였다. 이는 임상상황과 유사한 시나리오로 시뮬레이션 실습을 하는 동안 능동적 학습을 통해 직접 간호술기를 시행하고 환자에게 정보를 제공하는 과정에서 학습효과가 나타난 것이라 생각된다. 특히 본 연구에서는 시나리오 개발 시 Suh [26]가 제시한 시나리오 난이도 중 3단계인 상황특이적 상황 설정으로 간호사와 환자, 간호사와 간호사, 간호사와 타 의료인과의 의사소통을 통해 간호문제를 해결할 수 있도록 구성하였고, 운영과 적용 측면에서는 4–5명의 소그룹으로 15–20분 시뮬레이션 운영이 적절하다는 Bremner 등[27]의 연구에 근거하여 4–5명의 소그룹으로 시나리오 구현 15분, 디브리핑 40분으로 운영하였기에 임상수행능력 향상에 효과적이었다고 여겨진다.

비판적 사고성향의 경우 본 연구에서 실험군과 대조군 간에 통계적으로 유의한 차이가 없었는데, 이는 시뮬레이션 실습 후 간호학생의 비판적 사고능력이 향상되었다고 보고한 선행연구[23]와는 상반된 결과이나, 시뮬레이션 기반 교육과정 적용 후 실험군의 비판적 사고성향 점수가 대조군보다 증가하였으나 통계적으로 유의한 차이가 없었다고 보고한 선행연구[28]와는 유사하였다. 시스템 사고 통합 시뮬레이션 학습과 사례학습을 비교한 연구[28]에서 두 학습이 모두 비판적 사고성향을 증가시켰을 것이라는 분석처럼, 본 연구에서도 대조군의 사례연구가 비판적 사고성향 증가에 영향을 미쳐 시뮬레이션 실습을 한 실험군과 통계적으로 유의한 차이를 보이지 않았다고 생각해 볼 수 있다. 따라서 단지 시뮬레이션 실습을 통해서만이 아니라 간호학 교과목 전반에서 다양한 학습방법을 이용하여 비판적 사고성향을 학습하고 강화할 수 있는 교육체계를 마련할 필요가 있을 것이다.

본 연구에서 임상판단력은 실험군이 대조군에 비해 통계적으로 유의하게 향상되었다. 이는 간호대학생 3학년을 대상으로 시뮬레이션 기반 임상추론 실습교육 프로그램을 적용한 후 임상판단능력이 향상되었다는 연구결과[29]와 일치하였다. 본 연구에서는 1회의 중재에도 불구하고 실험군의 사전·사후 점수 차이는 17.41점, 대조군의 사전·사후 점수 차이는 11.07점으로, 간호대학 3학년 학생을 대상으로 5회의 시뮬레이션 교육 프로그램을 적용하여 임상판단력의 효과를 검증한 Lim [23]의 연구에서 보인 15.4점, 4.95점보다 높게 나타났다. 이는 평가자가 다르다는 제한점은 있으나, 임상판단력은 시뮬레이션 실습의 중재횟수에 크게 영향받지 않고 향상됨을 알 수 있다. 즉, 본 연구에서 사전학습을 통한 학습과제에 대한 이론적 지식을 바탕으로 대상자의 상태를 직접 사정하고 환자와 간호사, 간호사와 간호사, 간호사와 의사 간에 의사소통하면서 간호문제를 해결할 수 있도록 추가정보 등을 암시적으로 제공하여 바람직한 간호중재를 이끌어낼 수 있도록 시나리오 내용을 구성하였기 때문에 학생들이 임상적 판단과정을 통해 간호과정을 적용하면서 임상판단력이 향상되었다고 생각된다. 따라서 다양한 사례를 통해 간호학생의 문제해결능력과 임상적 추론능력을 향상시킬 수 있도록 시나리오를 구성하는 것이 중요할 것이다.

교육만족도의 경우 실험군이 대조군보다 만족도가 더 높은 것으로 나타났는데, 이는 간호대학 3학년 학생을 대상으로 시뮬레이션 기반 신생아 응급간호 교육 프로그램을 개발하여 그 효과를 검증한 연구[7]에서 실험군의 만족도가 유의하게 높게 나타난 결과와 유사하였다. 특히, 교육방법이 새롭고 효과적이며 교육 중에 받은 피드백이 도움이 되었다는 항목의 만족도가 높게 나타났으므로, 시뮬레이션 실습 시 즉각적인 처치가 필요한 위급상황이나 실제 임상상황에서 실습하기 어려운 내용을 다양하게 마련하고 충분한 피드백을 제공함으로써 학생들의 만족도를 높일 수 있도록 노력해야 할 것이다.

다만 본 연구는 비판적 사고성향 등 장기적인 중재효과를 고려해야 하는 변수의 효과를 추적 검증하지 못한 제한점이 있다. 그럼에도 불구하고 본 연구에서 개발한 산후출혈 산모 간호 시뮬레이션 교육 프로그램은 대상자의 임상수행능력과 임상판단력, 교육만족도의 향상에 효과가 있었다. 사전학습을 통해 배운 지식과 기술을 임상상황과 유사한 시뮬레이션 교육을 통해 종합적으로 실제 적용해 볼 수 있는 계기를 제공했다는 점, 전통적인 사례연구와 비교하여 그 효과를 검증하였다는 것과 여성건강간호학 실습자료로 활용될 수 있다는 점이 본 연구의 의의가 될 수 있을 것이다. 본 연구 결과를 통해 여성건강간호학 학습 내용 중 질환의 중요도가 높으나 실제 임상현장에서 실습이 어려운 다양한 내용에 대한 시나리오 개발이 이루어지길 기대하며, 시뮬레이션 실습 시 의료직종 간 협력이나 효율적인 의사소통을 강화할 수 있는 내용을 추가하여 지식뿐만 아니라 실제 임상현장에서 필요한 역량을 향상시킬 수 있는 프로그램을 개발할 것을 제언한다.

## Conclusion

본 연구를 통해 개발된 산후출혈 산모 간호 시뮬레이션은 교육 프로그램은 전통적인 실습교육방법인 사례연구와 비교하여 간호대학생의 임상수행능력과 임상판단력을 더욱 향상시키는 긍정적인 효과를 보였다. 시뮬레이션 기반 학습을 통해 실제 상황과 유사한 간호환경에서 실제 간호사로서 술기를 직접 수행하고 환자-간호사, 간호사-간호사, 간호사-의사 사이에 의사소통함으로써 임상수행능력과 임상판단력 및 비판적 사고성향 중 자신감 향상을 보여주었다. 또한 시뮬레이션이라는 새로운 실습교육 방법에 대한 기대감과 호기심으로 교육 후 학생들의 교육만족도 역시 사례연구를 한 대조군보다 훨씬 높아 매력적인 프로그램임을 확인하였다. 따라서 간호대학 3학년 학생들의 실습교육 시 산후출혈 산모 간호교육에 본 교육 프로그램을 이용한 시뮬레이션 실습을 적용한다면 임상에서 실제 경험하지 못한 산후출혈 산모 대상자 사례에 대한 효과적인 실습이 이루어질 것이며 학생들의 교육만족도도 높아질 것으로 생각된다.

## Figures and Tables

**Table 1.** Homogeneity of characteristics and variables between the experimental and control groups (N=64)

Variable	Categories	Experimental (n=33)	Control (n=31)	χ^2^/t	*p*
		Mean±SD or n (%)	Mean±SD or n (%)		
Age (year)		23.7±7.58	22.9±4.77	11.12	0.519
Gender	Man	0 (0)	3 (9.7)	3.35	.108^†^
	Woman	33 (100)	28 (90.3)		
Grade in women’s health nursing	A	6 (18.2)	11 (35.5)	3.99	0.262
	B	16 (48.5)	10 (32.3)		
	Below C	11 (33.3)	10 (32.2)		
Satisfaction with nursing		1.45±0.56	1.42±0.50	0.97	0.617
Level of self-expression		1.73±0.57	1.77±0.72	2.33	0.312
Human relationships		1.52±0.50	1.58±0.50	0.28	0.599
Clinical performance		5.45±3.86	6.52±4.76	0.98	0.329
Critical thinking disposition		97.18±9.09	98.35±10.35	0.48	0.632
Clinical judgment		18.97±5.67	19.32±6.97	0.22	0.824

† Fisher exact test.

**Table 2.** Comparison of variables and learning satisfaction between groups (N=64)

Variable	Categories	Experimental (n=33)	Control (n=31)	t	*p*
		Mean±SD	Mean±SD		
Clinical performance	Pre	5.45±3.86	6.52±4.76	–4.80	<.001
	Post	21.38±2.87	16.05±3.49		
	Post-pre	15.92±4.09	9.53±5.23		
Critical thinking disposition	Pre	97.18±9.09	98.35±10.35	–1.68	0.097
	Post	98.42±11.06	96.77±11.67		
	Post-pre	1.24±5.82	–1.58±7.53		
Clinical judgment	Pre	18.97±5.67	19.32±6.97	–4.14	–4.14
	Post	36.38±2.55	30.39±4.39		
	Post-pre	17.41±5.32	11.07±6.89		
Learning satisfaction	Post	23.97±2.70	16.45±3.05	–10.45	<.001
